# A new selective culture medium for isolation of *Burkholderia cepacia* complex in pharmaceutical industry

**DOI:** 10.3389/fmicb.2025.1631983

**Published:** 2025-08-29

**Authors:** Meng Yu, Sijin Wang, Yao Zhong, Li Yuan, Likang An, Danyang Feng, Zhen Liu, Shihong Ma

**Affiliations:** ^1^National Institutes for Food and Drug Control, Beijing, China; ^2^Liaoning Institute for Drug Control, Shenyang, China; ^3^Henan Institute for Drug and Medical Device Inspection, Zhengzhou, China; ^4^Hebei Institute for Drug and Medical Device Control, Shijiazhuang, China; ^5^Shandong Institute for Food and Drug Control, Jinan, China; ^6^Zibo Institute for Food and Drug Control, Zibo, China

**Keywords:** growth-promoting, selectivity, indicative property, *Burkholderia cepacia* complex, objectionable microorganisms, selective medium

## Abstract

**Objectives:**

*Burkholderia cepacia* complex (Bcc) are typical objectionable microorganisms of concern for water-based pharmaceuticals. In order to achieve quick and effective detection of Bcc to prevent final product contamination, a new selective medium, *Burkholderia cepacia* complex selective agar (BCCSA), for the detection of Bcc in water-based pharmaceutical products was reported.

**Methods:**

The formulation of BCCSA was optimized based on the carbon sources utilization of 60 Bcc strains from multiple origins. BCCSA and *Burkholderia cepacia* selective agar (BCSA), which was adopted in the examination chapter for Bcc in United States Pharmacopoeia (USP), were compared in terms of growth-promoting, indicative and inhibitory properties using Bcc and non-Bcc strains. In addition to streaking and spreading on solid media, this study determined the growth curves of Bcc strains in liquid culture systems to discuss the growth-promoting ability of the two selective media from both qualitative and quantitative perspectives. 168 strains of non-Bcc were streaked onto the two media to compare inhibitory capability.

**Results:**

*α*-D-Lactose was unable to be utilized by all 60 test Bcc strains, while Sucrose was used by some Bcc strains, hence the carbon source composition of the medium BCCSA was adjusted by replacing lactose with sodium pyruvate and reducing the amount of sucrose added. The initial pH value of the medium was set as 6.2 ± 0.2 at 25°C by adding potassium dihydrogen phosphate, taking into account the indication range of phenol red. The recovery of 16 Bcc standard strains on BCCSA showed no statistically significant difference compared to TSA (*p* = 0.68), whereas BCSA demonstrated a significant reduction (*p* = 0.03). When the experimental scope was extended to 40 strains mainly pharmaceutical related, BCCSA recovered a higher ratio (82% at 24 h incubation, 97% at 48 h) than BCSA (67% at 24 h, 90% at 48 h). It has been confirmed that the test Bcc strains exhibited higher growth rates in BCCSA by growth curve analysis (*p* = 0.02). For 168 non-Bcc strains, BCSA inhibited 94% of non-Bcc growth, while BCCSA inhibited 90%, with no significant difference.

**Conclusion:**

This novel medium BCCSA demonstrates enhanced efficiency and comparable selectivity in detecting Bcc in contrast to BCSA, rendering it more suitable for risk identification of Bcc in samples during pharmaceutical manufacturing process.

## 1 Introduction

*Burkholderia cepacia* complex (Bcc) is a group of multidrug-resistant organisms, that are also resistant to many disinfectant cleansers and antimicrobial preservatives (Lee et al., 2013; Song et al., 2018), because of various mechanisms (Mahenthiralingam et al., 2005). Bcc is a collection of genetically distinct but phenotypically similar bacteria that are divided into at least 27 species, which are commonly found in natural environments such as soil, water, and agriculture products (Jin et al., 2020; Morales-Ruíz et al., 2022; Velez et al., 2023). As opportunistic pathogens, Bcc are capable of causing disease primarily among patients of Cystic Fibrosis (CF) and immunocompromised populations (Landes et al., 2016; Lyczak et al., 2002). Now Bcc are widely noted as typical objectionable microorganisms for water-based non-sterile pharmaceutical products in the pharmaceutical industry (Parenteral Drug Association, 2014) as they may pose serious consequences to vulnerable patients. Numerous non-sterile products are recalled due to microbiological contamination every year, with Bcc accounting for approximately 50% of these cases (Jimenez, 2007; Sutton and Jimenez, 2012). U. S. Food and Drug Administration (FDA) advised drug manufacturers that Bcc poses a contamination risk in non-sterile, water-based drug products for twice in 2017 and 2021, respectively (FDA, 2023). Meanwhile, FDA issued guidance related to microbiological quality considerations of non-sterile products, in which risk identification and control of Bcc contamination for products was emphasized (U.S. Food and Drug Administration, 2025). Preventing Bcc contamination in drugs by addressing the potential sources in a drug manufacturing process is an important public health goal.

Since the early 1980s, researchers have begun to develop selective media for Bcc isolation. In 1985, a selective medium for isolation of *B. cepacia* was developed, named Pseudomonas cepacia Agar (PCA)(Gilligan et al., 1985). Subsequently their formulation was improved by adjusting the content of carbon and nitrogen sources of inhibitory substances, and lowering the initial pH of the medium to form the commercialized Bcc selective culture medium Mast *Burkholderia Cepacia* Agar (BCA) and Oxoid BCA. In 1987, antibiotics were added for screening on the basis of lactose oxidative fermentation medium to form the selective medium Oxidation Fermentation Polymyxin Bacitracin Lactose (OFPBL) Agar for *B.cepacia* (Welch et al., 1987). In 1997, Henry et al. improved this medium by adding sucrose, yeast extract, increasing the content of tryptone, adjusting the type and concentration of antibiotics, and finally forming the *Burkholderia Cepacia* Selective Agar (BCSA) (Henry et al., 1997).

For non-sterile pharmaceutical products, the United States Pharmacopeia (USP) first incorporated the examination method for Bcc in 2019, as outlined in General Chapter <60> (USP Convention, 2021). BCSA was used for selection and subculture of Bcc after bacterial enrichment. In the process of developing the Bcc examination method for the Chinese Pharmacopoeia (ChP), comprehensive studies were conducted on selective agars to identify a medium that is more suitable for detecting samples within the pharmaceutical industry. As one of the outcomes of this study, the new *Burkholderia cepacia* Complex selective agar (BCCSA) was developed. Bcc strains from international collection and industrial production-related strains were used to compare the growth-promoting property of BCCSA with BCSA. Meanwhile, the two selective media were challenged by 168 strains from drugs and water-system of selectivity ability. This paper reports the optimization process to BCSA and the basic characteristics of BCCSA.

## 2 Materials and methods

### 2.1 Culture media

The components of BCSA and BCCSA are shown in Table 1. BCSA (product ref:11317) and supplement (product ref: 11317a) was obtained from Beijing SanYao Science & Technology Co., Ltd, Beijing, China, and prepared in accordance with manufacturer’s instructions. The composition (per liter of distilled water) of BCCSA is as follows: sucrose 2.0 g, sodium pyruvate 7.0 g, trypticase peptone 10.0 g, NaCl 5.0 g, yeast extract 1.5 g, KH_2_PO_4_ 1.54 g, phenol red 0.02 g, crystal violet 0.001 g, agar 14.0 g, polymyxin B sulphate 600 000 IU, gentamicin 0.01 g, vancomycin 0.0025 g. The phenol red and crystal violet were prepared as 0.2% and 0.01% aqueous solutions, respectively, and 10 ml of each was added per liter. After autoclaving for 15 min at 121°C, antibiotics were added when the medium is cooled to around 50°C. Soybean-Casein Digest Agar (TSA, ref: 236950), Soybean-Casein Digest Broth (TSB, ref: 211825) and Sabouraud Dextrose Broth (SDB, ref: 238230) was obtained from Becton-Dickinson, Rungis, France.

**Table 1:** Formula of BCSA and BCCSA.

Component category	BCSA ingredients (per litre)		BCCSA ingredients (per litre)	
Carbon source	Surcose	10.0 g	Surcose	2.0 g
	Lactose	10.0 g	Sodium pyruvate	7.0 g
	Yeast extract	1.5 g	Yeast extract	1.5 g
Nitrogen source	Casein peptone	10.0 g	Trypticase peptone	10.0 g
Osmotic regulator	Sodium chloride	5.0 g	Sodium chloride	5.0 g
Buffering agent			Potassium dihydrogen phosphate	1.54 g
pH indicator	Phenol red	0.08 g	Phenol red	0.02 g
Agar		14.0 g		14.0 g
Selective agent	Crystal violet	0.002 g	Crystal violet	0.001 g
	Gentamicin	0.01 g	Gentamicin	0.01 g
	Vancomycin	0.0025 g	Vancomycin	0.0025 g
	Polymyxin B	600,000 IU	Polymyxin B	600,000 IU
Final pH (at 25°C)	6.8 ± 0.3		6.2 ± 0.2	

### 2.2 Bacterial strains

Twenty strains of Bcc and eleven strains of non-Bcc were purchased separately from China Center of Industrial Culture Collection (CICC), China National Center for Medical Culture Collections (CMCC), China General Microbiological Culture Collection Center (CGMCC) and American Type Culture Collection (ATCC). A further 40 strains of Bcc and 157 strains of non-Bcc were retrieved from water-based pharmaceutical, cosmetic products and pharmaceutical water systems. In total, 60 strains of Bcc were tested including: *Burkholderia cepacia* (*n* = 24), *Burkholderia aenigmatica* (*n* = 13), *Burkholderia cenocepacia* (*n* = 11), *Burkholderia contaminans* (*n* = 4), *Burkholderia vietnamiensis* (*n* = 3), *Burkholderia stabilis* (*n* = 2), *Burkholderia multivorans* (*n* = 1), *Burkholderia pyrrocinia* (*n* = 1), *Burkholderia ambifaria* (*n* = 1).

Bacterial strains used in this study were identified through the analysis of the 16S rRNA gene. The 16S rRNA gene was amplified using the primers 27f (5′-AGAGTTTGATCCTGGCTCAG-3′) and 1492r (5′-TACGGCTACCTTGTTACGACTT-3′). The PCR reaction mixture (50 μL) contained: Taq DNA polymerase (5 U/μL) 0.25 μL, upstream and downstream primers (10 μmol/L) 1 μL each, 10 × PCR buffer 5 μL, dNTP mixture (each 2.5 mmol/L) 4 μL, DNA template 1 μL, and ddH₂O to adjust the volume to 50 μL. The PCR reaction conditions were as follows: initial denaturation at 95°C for 4 min; 35 cycles of denaturation at 95°C for 30 s, annealing at 50°C for 45 s, and extension at 72°C for 1 min and 30 s; final extension at 72°C for 5 min. Bcc strains were additionally identified by recA gene sequence analysis. The *recA* gene was amplified using the primers BCR1 (5′-TGACCGCCGAGAAGAGCAA-3′) and BCR2 (5′-CTCTTCTTCGTCCATCGCCTC-3′) (Mahenthiralingam et al., 2000). The reaction mixture was identical to that used for the 16S rRNA gene amplification. The PCR reaction conditions were as follows: initial denaturation at 94°C for 3 min; 32 cycles of denaturation at 94°C for 30 s, annealing at 56°C for 45 s, and extension at 72°C for 1 min; final extension at 72°C for 10 min. Fungi used in this study were identified through colony observation, microscopic examination, and Internal Transcribed Spacer (ITS) sequence analysis. The ITS gene was amplified using the primers ITS F (5′-GGAAGTAAAAGTCGTAACAAGG-3′) and ITS R(5′- TCCTCCGCTTATTGATATGC-3′). The reaction mixture was identical to that used for the 16S rRNA gene amplification. The PCR reaction conditions were as follows: initial denaturation at 94°C for 10 min; 29 cycles of denaturation at 94°C for 30 s, annealing at 55°C for 30 s, and extension at 72°C for 30 s; final extension at 72°C for 5 min. The amplified products were sequenced using the Sanger sequencing method, performed by Beijing Novogene Bioinformatics Technology Co., Ltd. The sequences were compared and analyzed using BLAST (https://blast.ncbi.nlm.nih.gov/Blast.cgi). In addition to sequence analysis methods, Bcc strains were identified by the Omnilog system on the basis of utilization of carbon sources, which is described in section 2.3.

### 2.3 Biolog: carbon source utilization test of Bcc

A 96-well automated Omnilog system with GENIII Micro plates (Biolog Inc., Hayward, CA, USA) was used for the characterisation of carbon source utilization. Microplates were set up and analyzed following the manufacturer’s instructions. Single colonies of 60 strains of Bcc were subcultured on TSA plates separately. After 24 h incubation at 33°C, colonies were collected with a cotton swab and suspended in 18 mL in oculation fluid A (IF-A, Catalog No.72401) to obtain a homogeneous suspension. The turbidity of the bacterium suspensions was set to 90 to 98% using the Biolog Turbidimeter. GENIII MicroPlates were inoculated with 100 μL bacterium suspension per well and incubated at 33°C. The OmniLog system includes an incubator and a charged-coupled device camera system. The OmniLog digital camera measures the color level of each well in OmniLog Units (OU). Tetrazolium redox dyes indicate the extent of microbial utilization of carbon sources and sensitivity to chemicals through changes in color intensity. A positive result is indicated by the reaction wells turning purple, while a negative result is indicated by the reaction wells remaining colorless. Metabolic activity was determined by the system at 15-min intervals automatically during a whole 36 h incubation. The metabolic results were shown by Omnilog software (Biolog Inc., USA, Version 4.20.05). By comparing the carbon source utilization profile of the tested strain with those in the database, Omnilog system provided an identification result at the species level.

### 2.4 Quantitative recovery of pure Bcc strains

Quantitative recovery of Bcc on selective media was assessed with four strains: ATCC 25416 = CICC10857 contained in USP (general chapter <60>), CMCC(B)23005, CMCC(B)23006, CMCC(B)23010 contained in the ChP (General chapter 1109). They were subcultured in TSB at 33°C for 18 to 24 h, then performed by adding 100 μL of suspension to 0.9 mL of 0.9% NaCl solution for serial tenfold dilutions. For each strain, an inoculum of approx. 10^3^ CFU/mL was intended to be obtained. A 100 μL aliquot of each suspension was spread on the two selective agar plates, as well as TSA. For each suspension, two petri dishes were used. Colonies were counted after incubation at 33°C for 48 to 72 h. Each strain was subjected to 10 experimental replicates. TSA is a non-selective medium and served as a control. The ratio of recovery (%) was determined by colony-forming unit (CFU) counted on selective media to that on TSA for each strain.

To further expand the scope of investigation, the recovery assay was also conducted using the additional sixteen standard strains employed in this study. Strains were inoculated into TSB individually and incubated for approximately 24 h to achieve an initial bacterial concentration of around 10^8^ CFU/mL. Subsequently, serial 10-fold dilutions of the cultures were performed using 0.9% NaCl solution, with the original culture defined as dilution level 0. From each dilution series, 100 μL of the 10^−5^ and 10^−6^ dilutions was spread onto TSA, BCSA and BCCSA plates using a spreader, with two replicate plates for each dilution. The plates were then incubated at 33°C for 48 h, after which the number of CFU was recorded. The recovery assay for each strain was repeated three times.

### 2.5 Growth of Bcc on selective media

A total of 60 Bcc strains were subcultured in TSB at 33°C for 18 to 24 h. An inoculum with an approximate concentration of 10^6^ to 10^8^ CFU/mL was preparared from these cultures. About 10 μL of each dilution was performed three-zone streaking inoculation on different selective media using a disposable inculating loop. The inoculated plates were incubated at 33°C for 24 and 48 h. The plates were examined at both time points to assess bacterial growth. After of incubation. The number of strains that grew in the main inoculum area, the second quadrant and the third quadrant were recorded at each observation time point. This approach allows for a detailed assessment of the distribution and growth dynamics of the Bcc strains across different zones of the streaked plates.

### 2.6 Growth curve experiments

Fifteen strains of Bcc were initially cultured in TSB at 33°C for 24 h to obtain a uniform and active culture. An inoculum with an approximate concentration of 10^6^ CFU/mL was prepared by appropriate dilution of the overnight culture. BCSA and BCCSA were prepared as liquid media according to their respective formulations, excluding agar, phenol red, and crystal violet. These components were omitted to facilitate optical density measurements without interference from solidifying agents or pH indicators. For the automated growth curve analysis, 20 μL of the prepared inoculum was mixed with 180 μL of the liquid culture medium (either BCSA or BCCSA) and transferred into the wells of a Bioscreen plate (Labsystems, Helsinki, Finland). Each condition was tested in quadruplicate to ensure statistical robustness. Control wells contained only the culture medium without inoculum. The Bioscreen C system (Labsystems, Helsinki, Finland) was used for automated growth curve analysis. The instrument was preheated to 33°C to ensure consistent temperature conditions throughout the experiment. The absorbance of the cell suspensions at 600 nm was measured every 10 min over a period of 72 h. Prior to each measurement, the cultures were automatically agitated for 15 s to ensure uniform distribution of cells.

The absorbance data obtained from the Bioscreen C system were exported to Microsoft Excel 2010 for further processing. The average absorbance values for each type of culture medium were calculated from the quadruplicate measurements. Growth curves were generated by plotting the average OD600 values against incubation time (in hours). To eliminate the influence of medium color on the absorbance readings, the OD600 values of the experimental wells were corrected by subtracting the corresponding values from the control wells. The maximum specific growth rate (*μ*_max_) for each strain in different media was determined by fitting the logarithmic phase of the growth curves, ensuring that the *R*^2^ value was ≥0.99.

### 2.7 Evaluation of selective media with non-Bcc strains

Non-Bcc bacterial strains (*n* = 156) were subcultured in TSB at 33°C for 18 to 24 h. Yeasts (*n* = 10) were subcultured in SDB at 23°C for 24 to 48 h. The spore suspensions of filamentous fungi (*n* = 2) were prepared and stored by our laboratory. An inoculum with an approximate concentration of 10^6^ to 10^8^ CFU/mL was preparared from bacterial cultures. An inoculum with an approximate concentration of 10^5^ CFU/mL was preparared from fungal cultures. About 10 μL of each dilution was performed three-zone streaking inoculation on different selective media using a disposable inculating loop. The inoculated plates were incubated at 33°C for 72 h. A strain was recorded as “growing” by colonies present in the main inoculum area.

### 2.8 Statistical analysis

Difference in performance of recovery for each Bcc strain of the two media was investigated for statistical significance using Students’ test. Difference in performance of growth curves of the two media was investigated using McNemar’s test with the continuity correction applied. Statistical significance was assigned to a probability (*P*) value of < 0.05. For the sixteen other standard strains, a generalized linear mixed model (GLMM) with a negative binomial distribution (nbinom2 parameterization) was fitted to assess the effects of media type on bacterial colony counts, while accounting for between-strain variability. The model was specified as:


count∼media+(1∣strain)

Where: fixed effect: media (categorical: BCSA, BCCSA, TSA as control); random intercept: strain (16 levels) to model subject-specific variability; distribution: negative binomial (log link) to handle overdispersion (variance > mean). Model fitting was performed using the glmmTMB package (v1.1.7) in R (v4.5.1). Significance of fixed effects was evaluated via Wald *z*-tests with *α* = 0.05. Residual diagnostics of the model were conducted using the DHARMa package (v0.4.7).

## 3 Results

### 3.1 Optimization of carbon sources of the BCSA based on utilization of Bcc strains

Table 2 provides an overview of the carbon source utilization patterns of Bcc strains on the Omnilog GenIII reaction plate. The results indicated that several carbon sources, including D-Maltose, D-Cellobiose, D-Pine Disaccharide, α-D-Lactose, L-Rhamnose, Inosine and D-Lactic Methyl Ester were not utilized by any of the tested Bcc strains. In contrast, carbon sources such as α-D-Glucose, D-Galactose, L-Fucose and Glucuronide were utilized by the strains, as evidenced by positive reactions. Notably, *B. ambifaria* (*n* = 1), which differed from other Bcc strains, was unable to utilize certain carbon sources including D-Mannose, D-Fructose, D-glucose-6-Phosphate, D-Fructose-6-phosphate, D-Gluconic Acid and L-Malic Acid. This finding was consistent with the description in Berger’s Manual of Systematic Bacteriology,which states that Bcc strains are unable to utilize α-D-Lactose (Garrity et al., 2005). This inability to utilize α-D-Lactose was a crucial factor in the formulation adjustment of BCSA. Based on the carbon utilization characteristics of Bcc, lactose in BCSA was replaced with sodium pyruvate, and the content of sucrose was adjusted to develop a new selective culture medium BCCSA.

**Table 2:** Carbon source utilization of 60 Bcc strains.

Carbon sources	Species								
	*Burkholderia cepacia*	*Burkholderia cenocepacia*	*Burkholderia stabilis*	*Burkholderia multivorans*	*Burkholderia ambifaria*	*Burkholderia vietnamiensis*	*Burkholderia pyrrocinia*	*Burkholderia contaminans*	*Burkholderia aenigmatica*
D-Maltose	−	−	−	−	−	−	−	−	−(10), B(3)
D-Alginose	B(7), −(17)	+(8), −(3)	−	B	−	−	−	+(1), B(2), −(1)	−(9), B(4)
D-Cellobiose	−	−	−	−	−	−	−	−	−
Gentian disaccharide	+(2), B(2), −(20)	B(1), −(10)	−(1), B(1)	B	−	−	−	−	−(10), B(3)
Sucrose	+(17), −(4)	+(9), B(1), −(1)	−	−	−	+	−	+	+(8), B(4), −(1)
D-Pine Disaccharide	−	−	−	−	−	−	−	−	−(9), B(4)
α-D-Lactose	−	−	−	−	−	−	−	−	−
β-Formyl-D-glucoside	−	−	−	−	−	−	−	−	−(12), +(1)
D-Salicin	+(2), B(15), −(7)	+(9), −(2)	−	−	−	−	−	+(1),−(3)	+(4), B(4), −(5)
N-Acetyl−D−glucosamine	+(20), B(1), −(3)	+(9), B(1), −(1)	−(1), B(1)	+	−	+(1), B(2)	−	+(1), B(1), −(2)	+(6), B(4), −(3)
α-D-Glucose	+(22), −(2)	+(10), B(1)	+	+	+	+	+	+	+
D-Mannose	+(19), B(2), −(3)	+(10), −(1)	+	+	−	+(1), B(2)	B	+(3), B(1)	+(8), B(3), −(2)
D-Fructose	+(22), −(2)	+(10), −(1)	+	+	−	+(2), B(1)	+	+(3), B(1)	+(8), −(2)
D-Galactose	+	+(10), B(1)	+	+	+	+	+	+	+
3-Formylglucose	−	+(2), −(9)	−(1), B(1)	−	−	−	−	−	−
D-Fucose	+(2), B(6), −(16)	+(2), B(1), −(8)	−(1), B(1)	−	−	+(1), B(2)	−	B	+(2), B(6), −(5)
L-Fucose	+(23), B(1)	+(10), −(1)	+	+	+	+(1), B(2)	+	+	+(12), B(1)
L-Rhamnose	+(5), −(19)	B(1), −(10)	−(1)	−	−	B(1), −(2)	−	−	−
Inosine	B(1), −(23)	−	−	−	−	−	−	−	−
D-Sorbitol	+(17), B(4), −(3)	B(9), −(2)	+(1), −(1)	B	−	+(1), B(2)	−	+(3), B(1)	+(9), B(2), −(2)
D-Mannitol	+(17), B(4), −(3)	+(8), B(1), −(2)	+(1), −(1)	B	−	+(1), B(2)	−	+(3), −(1)	+(7), B(3), −(3)
D-Arabinol	+(12), B(7), −(5)	+(9), −(2)	+(1), −(1)	B	−	B(2), −(1)	−	+(2), B(1), −(1)	+(2), B(4), −(7)
D-Glucose-6-phosphate	+(22), −(2)	+(10), −(1)	+	+	−	+	+	+	+(12), B(1)
D-Fructose-6-phosphate	+	+(10), B(1)	+	+	−	+	+	+	+
D-Aspartic acid	+(13), −(11)	−	+(1), −(1)	−	−	−	−	+(3), B(1)	−
D-Serine	+(21), −(3)	−	+(1), −(1)	−	−	−	−	+(3), B(1)	+(10), B(2), −(1)
L-Alanine	+(20), B(2), −(2)	+(10), B(1)	+(1), −(1)	+	−	+(2), B(1)	−	+	+(11), B(1), −(1)
L-Arginine	+(20), −(4)	+(9), −(2)	+(1), −(1)	+	−	+(2), −(1)	−	+	+(11), B(1), −(1)
L-Aspartic acid	+(19), B(2), −(3)	+(9), −(2)	+(1), −(1)	+	−	+(2), B(1)	−	+(3), B(1)	+(6), B(4), −(3)
L-Glutamic acid	+	+(10), B(1)	+(1), −(1)	+	−	+	B	+	+(12), −(1)
L-Serine	+(16), B(5), −(3)	+(9), B(1), −(1)	+(1), B(1)	+	−	+(2), B(1)	−	+(3), B(1)	+(9), B(3), −(1)
D-Galacturonic acid	+(22), B(2)	+(10), −(1)	+	−	+	+(2), B(1)	+	+	+(9), B(4)
L-Galacturonic acid lactone	+(13), B(4), −(7)	+(3), B(1), −(7)	B(1), −(1)	−	B	+	+	+(3), B(1)	+(11), B(1), −(1)
D-Glucuronic acid	+(20), B(2), −(2)	+(9), B(2)	+	+	−	+	+	+(4)	+
D-Glucuronic acid	+(23), B(1)	+(10), B(1)	+	+	+	+(2), B(1)	+	+(4)	+(10), B(3)
Glucuronide	+(15), B(9)	+(9), B(2)	+(1), B(1)	+	+	+	+	+(3), B(1)	+(11), B(2)
Methyl pyruvate	+(4), B(11), −(9)	+(7), B(1), −(3)	+(1), −(1)	+	−	+(2), B(1)	−	+(2), B(2)	+(4), B(3), −(6)
Methyl D-lactate	−	−	−	−	−	B(1), −(2)	−	−	−
L-Lactic acid	+(21), −(3)	+(2), B(2), −(7)	+(1), −(1)	−	−	+	B	+	+(11), B(1), −(1)
α-Ketoglutaric acid	B(1), −(23)	B(1), −(10)	+(1), −(1)	−	−	B(1), −(2)	−	−	−
D-Malic Acid	B(6), −(18)	B(2), −(9)	+(1), −(1)	−	−	+(1), B(1), −(1)	−	+(1), B(3)	+(2), −(11)
L-Malic acid	+(22), −(2)	+	+	+	−	+	+	+	+

“+” indicates positive, “−” indicates negative, and “B” indicates indeterminate value. In the GenIII microplate, the carbon source utilization tests in columns 1–9 are referenced against the A-1 negative control well for colorimetric comparison. Wells that visually appear similar to the A-1 well are designated as “negative” (−). Wells that clearly display a purple color (darker than A-1) are designated as “positive” (+). Wells with very faint coloration, small purple spots, or the presence of bacterial clumps or other particulate matter should be classified as “indeterminate”.

The content of phenol red in BCCSA is 0.02 g/L, and the content of crystal violet is 0.001 g/L. Given that the pH indication of phenol red ranges from 6.8 to 8.4, the initial pH value of BCCSA was adjusted to approximately 6.2 using dipotassium hydrogen phosphate as the pH regulator, resulting in a yellow coloration of the agar. During growth, peptone in BCCSA is utilized by Bcc as a nitrogen source, producing ammonia via deamination or urease activity. The hydrolysis of ammonia to form hydroxide ions leads to an increase in the pH of the medium, which in turn induces a noticeable shift in the medium’s color from yellow to red. This color change facilitates convenient and clear observation by experimenters.

### 3.2 Growth of Bcc strains on BCSA and BCCSA

**Figure 1:**
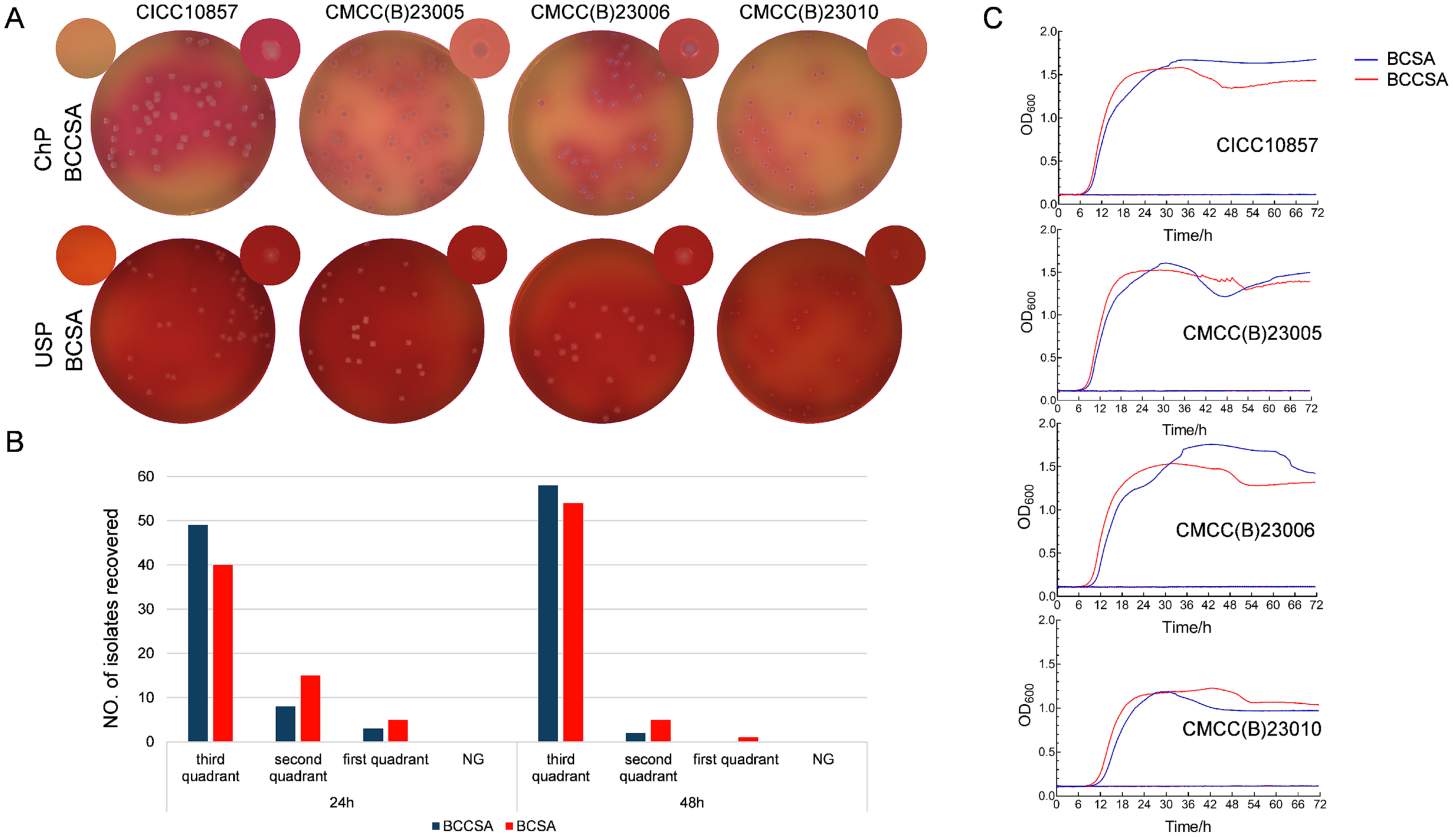
Growth of Bcc strains on BCSA and BCCSA. **(A)** The appearance and indication reactions of 4 Bcc reference strains on the two selective media after 72 h incubation. **(B)** The recovery of 60 Bcc strains on the two selective media after 24 to 48 h incubation. No. of strains grown only in first quadrant of the main inoculum area; grown into the second quadrant; grown into the third quadrant, and with no growth (NG) were recorded. **(C)** The growth curves of 4 Bcc reference strains in the liquid culture system of BCSA and BCCSA.

Table 3 showed the recovery rate results of the four standard strains commonly employed as reference strains in pharmaceutical quality control tests, on the two selective media. For each strain, the recovery rate was calculated as the ratio of CFU on each selective medium to those on TSA. Each strain was tested in 10 replicates. For both media, recovery rates of 4 strains satisfied a factor ranging from 0.5 to 2. The recovery rate on BCCSA was significantly higher compared to both BCSA of CICC10857 = ATCC25416 (*p* < 0.01, Student’s *t* test) and CMCC(B)23010 (*p* < 0.01). The differences in recovery rate between the two selective media were not found to be statistically significant for an additional two strains. Figure 1A showed the appearance and indication reactions of the four strains on the two media at 72 h of incubation. Delayed growth on BCSA was manifested in smaller colonies than BCCSA.

**Table 3:** Recovery of 4 Bcc strains on BCSA and BCCSA.

Strains	% Recovery^a^		*p* value
	BCSA	BCCSA	
CICC10857 = ATCC25416	77 ± 13	93 ± 8	*p*<0.01
CMCC(B)23005	88 ± 24	104 ± 20	NS
CMCC(B)23006	78 ± 17	88 ± 12	NS
CMCC(B)23010	61 ± 18	87 ± 22	*p*<0.01

^a^Each value caculated from ten times of test, and shown as the mean ± standard deviation. NS, not significant.

BCSA and BCCSA were evaluated using 60 strains, which included 20 strains from international collection and 40 strains isolated from pharmaceutical and cosmetic products. Recovery of Bcc strains on the two agar is illustrated in Figure 1B. After 24 h incubation at 33°C, all 60 strains were detected growth on both selective media. On BCCSA, 49 strains grew into the third quadrant of the inoculum area, 8 strains into the second quadrant, and 3 strains remained in the first quadrant. On BCSA, the distribution was 40 strains in the third quadrant, 15 in the second quadrant, and 5 in the first quadrant. After 48 hous of incubation, one strain isolated from oral liquid (YP20200287) still exhibited growth in the first quadrant with several colonies on BCSA. The number of strains that grew into the third quadrant on BCCSA and BCSA were 58 and 54, respectively.

**Figure 2:**
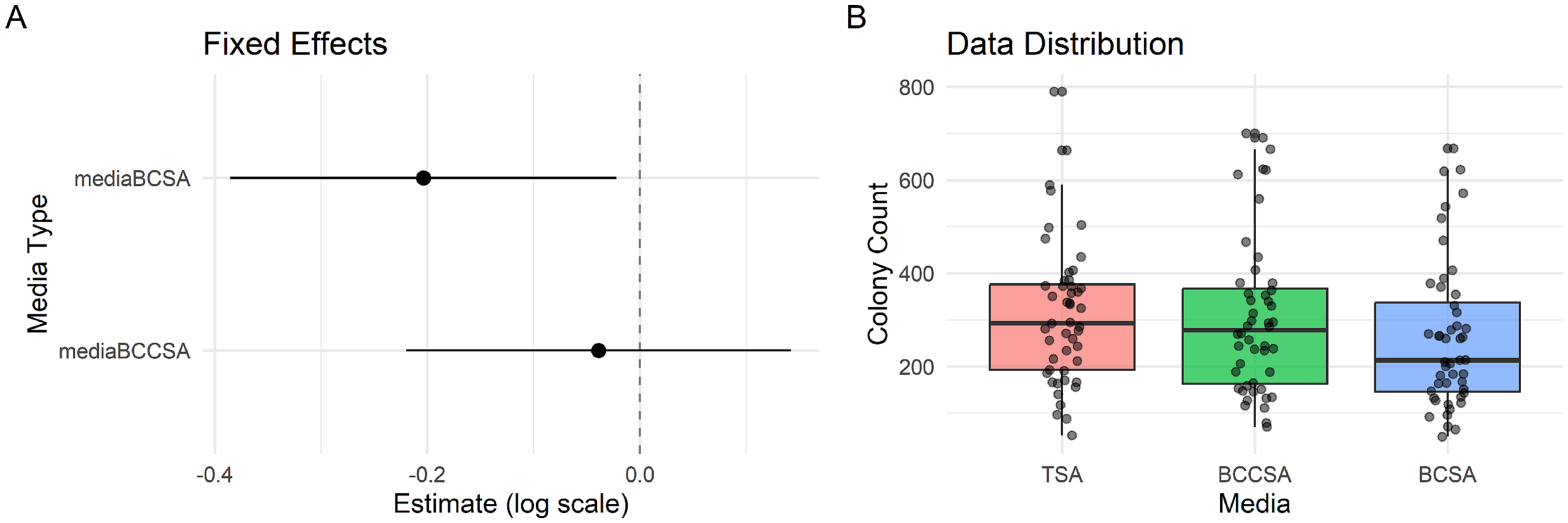
Results of sixteen Bcc strains recovered on BCSA and BCCSA by generalized linear mixed model (GLMM) with a negative binomial distribution (nbinom2 parameterization). **(A)** Forest plot displaying estimated effects of BCSA and BCCSA (reference: TSA) on Bcc colony counts (log10-transformed) with 95% confidence intervals. **(B)** Boxplots with overlaid jittered points showing raw colony count distributions across the three media types. Boxes represent the interquartile range (IQR), horizontal lines indicate medians, and individual points show observed data.

Figure 2B showed the original colony counts distribution of sixteen Bcc strains on three media. The overall effect of media was not statistically significant [χ^2^(2) = 5.43, *p* = 0.066]. However, post-hoc tests suggested a marginal reduction in colony counts for BCSA compared to the TSA (*β* = −0.203, SE = 0.093, *z* = −2.19, *p* = 0.028). BCSA showed a 18% reduction compared to TSA, with BCCSA did not show a significant difference to TSA (*β* = −0.039, SE = 0.092, *z* = −0.42, *p* = 0.675). The forest plot was shown as Figure 2A. Between-strain variability was modest (*σ^2^* = 0.098, SD = 0.314), indicating that 7.2% of variance (ICC) was attributable to strain differences after controlling for media effects. The model diagnostics were conducted using DHARMa’s simulated residuals approach, which confirmed the adequacy of the negative binomial GLMM specification. Residual analysis showed no significant deviations from model assumptions, with the uniformity test (KS-test: *p* = 0.84) indicating proper distributional fit, the dispersion test (*p* = 0.87) confirming appropriate handling of overdispersion, and no evidence of problematic outliers (*p* = 0.38). Visual inspection of the Q-Q plot and residual-versus-fitted plot further supported these conclusions, as all diagnostic plots displayed the expected patterns without systematic deviations. The non-significant *p*-values (all > 0.05) collectively suggest that the model’s distributional assumptions were met and the random effects structure adequately accounted for between-strain variability. These results justify the use of inferential statistics from this model for drawing conclusions about media treatment effects.

### 3.3 Growth curves and *μ_max_* of Bcc in two liquid culture system

To accurately depict the dynamic growth of Bcc strains in both media, the automated growth curve determination method was employed to assess the growth curves of 15 Bcc strains in liquid selective media in this study. The inclusion of agar was omitted during the preparation process. Additionally, phenol red and crystal violet were excluded from the formulations due to their potential interference with OD value readings caused by changes in medium pH resulting from strain inoculation. Figure 1C showed growth curves of four of the 15 Bcc strains in liquid system of BCSA and BCCSA. The majority of microbiologists consider the “maximum specific growth rate” (*μ_max_*) as the measure of the growth intensity of an organism. Mathematically, *μ_max_* is defined as the slope of the (sigmoid) growth curve at its inflection point (Peleg and Corradini, 2011). Table 4 gives the *μ_max_* of 15 Bcc strains in the liquid systems of BCCSA and BCSA. The *μ_max_* in BCCSA was greater than that in BCSA for all strains except CGMCC23882 (*B. contaminans*) and CGMCC1.10511 (*B. ambifaria*). The differences were significant according to the McNemar’s test (*p* = 0.02).

**Table 4:** *μ_max_* of Bcc strains in two selective liquid system.

Test strains	*μ_max_* (*R*^2^*>0.99*)		*p* value
	BCCSA	BCSA	
CICC10857 = ATCC25416	0.0984	0.0841	0.02
CMCC(B)23005	0.0904	0.0801	
CICC23882	0.0654	0.0735	
CGMCC1.2872	0.0608	0.0517	
YP20190187	0.0798	0.0711	
YP20190199	0.0730	0.0616	
YP20190169	0.0740	0.0567	
YP20190176	0.0828	0.0703	
YP20190175	0.0811	0.0744	
CGMCC1.10511	0.0810	0.0809	
CGMCC1.3077	0.0673	0.0528	
YP20190177	0.0783	0.0699	
CGMCC1.3058	0.0763	0.0672	
CMCC(B)23010	0.0883	0.0739	
CMCC(B)23006	0.0649	0.0538	

### 3.4 The selectivity of two media for Bcc and other water-born microorganisms

Table 5 summarizes the growth performance of 168 non-Bcc strains evaluated for selectivity on BCSA and BCCSA. For the 168 strains tested, the two media had identical inhibition ability to Gram-positive bacteria and fungi, with one strain of *Bacillus altitudinis*, one strain of *Candida theae*, and one strain of *Wickerhamomyces anomalus* grown. 4 strains of *Ralstonia pickettii* and 2 strains of *Achromobacter xylosoxidans* grow well on both media. One strain of *Stenotrophomonas maltophilia* was detected weak growth with several colonies only on first quadrant of the inoculum area of both media. Difference mainly occurred in the media selectivity for strains from pharmaceutical water system, which accounted for 69% of the total number of strains tested. A single strain representing each of the taxonomic category of *Burkholderia gladioli*, *Phyllobacterium myrsinacearum*, *Sphingomonas leidyi*, *Pseudochrobactrum*, and 2 strains of *Sphingomonas paucimobilis* grow on BCCSA, while not on BCSA. Although there was no statistically significant difference in their ability to inhibit non-Bcc strains (*p* = 0.05), the nutrient formula of BCCSA seems to be more suitable for the growth of objectionable microorganisms from water system, which is more favorable for the pre-screening of contamination risks in water-based products.

**Table 5:** Summary of growth of 168 non-Bcc strains on BCCSA and BCSA following 72-hour incubation.

Organisms	No. of strains recovered on	
	BCCSA	BCSA
Gram-negative species	13	7
*Acinetobacter* species (*n* = 8)	0	0
*Burkholderia gladioli* (*n* = 1)	1	0
*Paraburkholderia* species (*n* = 3)	0	0
*Phyllobacterium myrsinacearum* (*n* = 6)	1	0
*Herbaspirillum* species (*n* = 3)	0	0
*Enterobacter* species (*n* = 9)	0	0
*Brevundimonas* species (*n* = 3)	0	0
*Pantoea* species (*n* = 2)	0	0
*Pseudomonas* species (*n* = 6)	0	0
*Chryseobacterium gambrini* (*n* = 2)	0	0
*Klebsiella* species (*n* = 4)	0	0
*Ralstonia pickettii* (*n* = 4)	4	4
*Citrobacter freundii* (*n* = 1)	0	0
*Erwinia* species (*n* = 1)	0	0
*Sphingomonas species* (*n* = 21)	3	0
*Sphingopyxis soli* (*n* = 1)	0	0
*Achromobacter xylosoxidans* (*n* = 3)	2	2
*Acidovorax soli* (*n* = 6)	0	0
*Psychrobacter pulmonis/faecalis* (*n* = 2)	0	0
*Novosphingobium species* (*n* = 2)	0	0
*Stenotrophomonas maltophilia* (*n* = 5)	1	1
*Escherichia coli* (*n* = 3)	0	0
*Pseudochrobactrum species* (*n* = 1)	1	0
*Ochrobactrum species* (*n* = 4)	0	0
*Methylorubrum populi* (*n* = 1)	0	0
*Pelomonas species* (*n* = 2)	0	0
*Cellulosimicrobium species* (*n* = 2)	0	0
Gram-positive species	1	1
*Bacillus altitudinis* (*n* = 1)	1	1
*Other Bacillus* species (*n* = 11)	0	0
*Brachybacterium muris* (*n* = 1)	0	0
*Deinococcus* species (*n* = 2)	0	0
*Brevibacterium* species (*n* = 5)	0	0
*Kocuria marina* (*n* = 1)	0	0
*Methylobacterium* species (*n* = 8)	0	0
*Microbacterium species* (*n* = 4)	0	0
*Mesorhizobium huakuii* (*n* = 2)	0	0
*Micrococcus* species (*n* = 5)	0	0
*Sporosarcina contaminans* (*n* = 1)	0	0
*Staphylococcus* species (*n* = 10)	0	0
Fungi and yeast	2	2
*Aspergillus niger* (*n* = 1)	0	0
*Aspergillus brasiliensis* (*n* = 1)	0	0
*Canidia albicans* (*n* = 2)	0	0
*Candida parapsilosis* (*n* = 1)	0	0
*Candida theae* (*n* = 1)	1	1
*Meyerozyma guilliermondii* (*n* = 3)	0	0
*Rhodotorula mucilaginosa* (*n* = 1)	0	0
*Torulaspora delbrueckii* (*n* = 1)	0	0
*Wickerhamomyces anomalus* (*n* = 1)	1	1
Total	16	10

## 4 Discussion

In this study, results of carbon source utilization tests revealed that *α*-D-Lactose could not be utilized by any of tested Bcc strains, whereas Sucrose was utilized by some Bcc strains. These findings suggest that these two disaccharides are not ideal carbon sources for Bcc growth. Therefore, the carbon source composition of the new medium BCCSA was modified by replacing lactose with sodium pyruvate (Gilligan et al., 1985). Additionally, the content of sucrose was reduced. The results of the growth promotion assays demonstrated that the modified formula was more suitable for the growth of Bcc strains. Specifically, Bcc standard strains from international collections exhibited poorer recovery on BCSA compared to BCCSA. Bcc strains exhibited both a lower rate of progression into the third quadrant on BCSA at 24 and 48 hours and slower maximum growth rates in liquid culture compared to BCCSA. Phenol red serves as a pH indicator in the two selective media, turning yellow under acidic conditions and red under alkaline conditions. In addition to phenol red, BCSA incorporates crystal violet, which not only inhibits Gram-positive bacteria but also adjusts the initial color of the medium. When the initial pH is set at 6.8 ± 0.3, the medium exhibits a brick-red color. The ammonia produced by the growth of Bcc strains leads to an increase in pH, shifting the color toward rose red, which is not significantly noticeable during observation. To enhance the medium’s indicative property, we reduced the concentrations of phenol red and crystal violet and adjusted the initial pH to a more acidic level (6.2 ± 0.2), resulting in a yellow color. Consequently, as the pH rises due to Bcc growth, the medium turns rose red, creating a more pronounced color difference that is easier to observe and assess. In terms of growth-promoting ability and indicative property, BCCSA is a more ideal selective medium for Bcc.

As Bcc are the main pathogens in patients with CF, most of current studies on selective media are based on clinical samples. Henry et al. (1999) used a large number of 656 specimens to evaluate BCSA, OFPBL and PCA. BCSA showed better sensitivity—100% compared to 96% for OFPBL and 84% for PCA, and significant advantages of selectivity. Vermis et al. (2003) tested 142 clinical and environmental strains, belonging to the nine genomovars of Bcc on BCSA, PCA, TB-T, *B. cepacia* LAB M, *B. cepacia* Mast and *B. cepacia* Oxoid, resulting a better recovery of 99% on BCSA and *B. cepacia* Mast than others. Marrs et al. (2021) compared BCSA, *B. cepacia* Mast and *B. cepacia* BD using 270 cough and sputum samples, there was a delayed detection of growth with a lower yield at 24 h on BCSA when compared with the other two media. Most of the subjects of these studies are clinical samples, focusing on evaluating the selectivity of the culture media, that is, the ability to inhibit non-Bcc strains, rather than considering the recovery effect of the culture media on environmental strains as the main evaluation parameter.

The growth of non-Bcc Gram-negative and positive strains was inhibited by a combination of polymyxin B sulfate, gentamicin, vancomycin, and crystal violet in BCCSA. When challenged with 168 non-Bcc strains, BCCSA possessed comparable selectivity as BCSA. Despite the identical system of inhibition, strains of *Burkholderia gladioli*, *Phyllobacterium myrsinacearum*, *Sphingomonas leidyi*, *Pseudochrobactrum*, and *Sphingomonas paucimobilis* were able to grow on BCCSA. Perhaps it is not solely the antibiotic that determines the growth of a strain, but also the precise nutritional composition.

Although not belonging to the Bcc group, the organisms above were kind of objectionable microorganisms like Bcc, presents risk for drugs and cosmetic (Moreira et al., 2005; Ryan and Adley, 2010), with clinical infection cases reported (Moreira et al., 2005; Coenye et al., 2003; Alkhatib et al., 2022) and non-sterile products recalls by the FDA (Sutton and Jimenez, 2012). It’s necessary to identify the risk of contamination as early as possible in the drug manufacturing process in order to safeguard the quality of the product and the safety of the patient’s medication.

## 5 Conclusion

The growth-promoting and inhibitory properties were compared between a novel selective medium—BCCSA, as reported in this study, and the previously established medium BCSA. Adjustment of carbon sources and initial pH value of the medium were performed to improve better growth-promoting and indicative properties. The BCCSA exhibited superior recovery rates for four international collection Bcc reference strains, with a significant difference observed for CICC10857 and CMCC(B)23010. Additionally, the recovery assay of 16 Bcc reference strains demonstrated a significant reduction in colony counts on BCSA compared to TSA (*p* < 0.05), while no statistically significant difference was observed between BCCSA and TSA. After 24 h of incubation, 82% of 60 Bcc strains recovered with growing into the third quadrant of the inoculum area on BCCSA, versus 67% on BCSA. After 48 h of incubation, the results were 97 and 90%, respectively. The BCCSA also demonstrated a significant advantage in terms of the maximum growth rate for 15 Bcc strains during the growth curves test (*p* = 0.02). For 168 non-Bcc strains isolated from multiple origins, two media exhibited comparable selectivity, while BCCSA demonstrated enhanced recovery of several high-risk strains associated with water-based pharmaceutical products due to its distinct carbon sources. The BCCSA was designated as the preferred medium for Bcc examination in general chapter 1109 of the 2025 edition of Chinese Pharmacopoeia.

## Data Availability

The raw data supporting the conclusions of this article will be made available by the authors, without undue reservation.
