# Correction: A new selective culture medium for isolation of *Burkholderia cepacia* complex in pharmaceutical industry

**DOI:** 10.3389/fmicb.2025.1695214

**Published:** 2025-10-08

**Authors:** Meng Yu, Sijin Wang, Yao Zhong, Li Yuan, Likang An, Danyang Feng, Zhen Liu, Shihong Ma

**Affiliations:** ^1^National Institutes for Food and Drug Control, Beijing, China; ^2^Liaoning Institute for Drug Control, Shenyang, China; ^3^Henan Institute for Drug and Medical Device Inspection, Zhengzhou, China; ^4^Hebei Institute for Drug and Medical Device Control, Shijiazhuang, China; ^5^Shandong Institute for Food and Drug Control, Jinan, China; ^6^Zibo Institute for Food and Drug Control, Zibo, China

**Keywords:** growth-promoting, selectivity, indicative property, *Burkholderia cepacia* complex, objectionable microorganisms, selective medium

There was a mistake in Table 1 as published. The dosage of vancomycin was mistakenly written as 0.025 g. The correct dosage is 0.0025 g. The corrected Table 1 appears below.

**Table 1:** Formula of BCSA and BCCSA.

**Component category**	**BCSA ingredients (per litre)**		**BCCSA ingredients (per litre)**	
Carbon source	Sucrose	10.0 g	Sucrose	2.0 g
	Lactose	10.0 g	Sodium pyruvate	7.0 g
	Yeast Extract	1.5 g	Yeast extract	1.5 g
Nitrogen source	Casein peptone	10.0 g	Trypticase peptone	10.0 g
Osmotic regulator	Sodium Chloride	5.0 g	Sodium chloride	5.0 g
Buffering agent			Potassium dihydrogen phosphate	1.54 g
pH indicator	Phenol red	0.08 g	Phenol red	0.02 g
Agar		14.0 g		14.0 g
Selective agent	Crystal violet	0.002 g	Crystal violet	0.001 g
	Gentamicin	0.01 g	Gentamicin	0.01 g
	Vancomycin	0.0025 g	Vancomycin	0.0025 g
	Polymyxin B	600 000 IU	Polymyxin B	600 000 IU
Final pH (at 25°C)	6.8 ± 0.3		6.2 ± 0.2	

A correction has also been made to the section 2 “**Materials and Methods**”, sub-section 2.1 “*Culture Media*”, Paragraph 1: “The components of BCSA and BCCSA are shown in Table 1. BCSA (product ref:11317) and supplement (product ref: 11317a) was obtained from Beijing SanYao Science & Technology Co., Ltd, Beijing, China, and prepared in accordance with manufacturer's instructions. The composition (per liter of distilled water) of BCCSA is as follows: sucrose 2.0 g, sodium pyruvate 7.0 g, trypticase peptone 10.0 g, NaCl 5.0 g, yeast extract 1.5 g, KH2PO4 1.54 g, phenol red 0.02 g, crystal violet 0.001 g, agar 14.0 g, polymyxin B sulphate 600 000 IU, gentamicin 0.01 g, vancomycin 0.0025 g. The phenol red and crystal violet were prepared as 0.2% and 0.01% aqueous solutions, respectively, and 10 ml of each was added per liter. After autoclaving for 15 min at 121 °C, antibiotics were added when the medium is cooled to around 50 °C. Soybean-Casein Digest Agar (TSA, ref: 236950), Soybean-Casein Digest Broth (TSB, ref: 211825) and Sabouraud Dextrose Broth (SDB, ref: 238230) was obtained from Becton-Dickinson, Rungis, France.”

The original version of this article has been updated.

